# Hydrophilic Tetraphenylethene-Based Tetracationic Cyclophanes: NADPH Recognition and Cell Imaging With Fluorescent Switch

**DOI:** 10.3389/fchem.2021.817720

**Published:** 2021-12-22

**Authors:** Dan Wu, Zhankui Zhang, Xinyang Yu, Bing Bai, Shaolong Qi

**Affiliations:** ^1^ College of Materials Science and Engineering, Zhejiang University of Technology, Hangzhou, China; ^2^ Key Laboratory of Bioorganic Phosphorus Chemistry and Chemical Biology, Department of Chemistry, Tsinghua University, Beijing, China; ^3^ Key Laboratory and Engineering Laboratory of Lymphatic Surgery Jilin Province, China-Japan Union Hospital of Jilin University, Changchun, China

**Keywords:** tetracationic cyclophanes, tetraphenylethene, NADPH recognition, host-guest complexation, fluorescent switch

## Abstract

A hydrophilic TPE-based tetracationic cyclophane TPE-cyc was synthesized, which could capture intracellular Nicotinamide adenine dinucleotide phosphate and fuel the antioxidative ability of tumor cells to detoxify reactive oxygen species (ROS). Meanwhile, upon the reduction by cellular GSH, TPE-cyc could light up tumor cells, acting as a GSH-responsive fluorescent switch to image cells with high resolution.

## Introduction

Understanding the significance of molecular recognition in complex biological processes where various nucleic acids, enzymes and nucleotides are involved, has helped chemists to develop intelligent structures with fascinating properties (Meyer et al., 2003; Wu et al., 2017; Wu D et al., 2021). Artificial molecular receptors specific to biomolecules have attracted increasing attentions in recent years owing to their potential applications in medical and biological fields (Jisha et al., 2006; Hariharan et al., 2007; Pluth and Raymond, 2007; Kuruvilla et al., 2008; Ojida et al., 2008; Jisha et al., 2010; Alfonso and Sola, 2020; Escobar and Pablo, 2021). Nicotinamide adenine dinucleotide phosphate (NADPH), a main cellular reductant, plays an important role in maintaining glutathione in its reduced modality (GSH), which eliminates intracellular reactive oxygen species (ROS), thus preventing cells from oxidative damage (Dröge, 2002; Ying, 2008; Celton et al., 2012; Schulze and Harris, 2012; Fernandez-Marcos et al., 2016). Although the probable mechanisms of NADPH-involved physiological processes have been proposed, deeper exploration is still needed due to the complexity and uncertainty of the existing mechanisms. Therefore, a molecular recognition system which can selectively recognize NADPH is urgently needed.

The discovery of crown ethers by Pedersen opened the way for supramolecular chemists to design macrocyclic molecules that act as molecular receptors based on non-covalent interactions or weak coordination (Pedersen, 1967). Since then, chemists have constructed various macrocyclic hosts such as cyclodextrins (Liao et al., 2010; Crini, 2014; Prochowicz et al., 2017; Chen et al., 2021), cucurbit [*n*]turils (Ong et al., 2002; Jeon et al., 2004; Lagona et al., 2005; Das and Scherman, 2011; Francisco et al., 2019), calix [*n*]arenes (Ikeda and Shinkai, 1997; Philip kaifer 2002; Sameni et al., 2009; Perret and Coleman, 2011; Li et al., 2020), cyclophanes (Ariga et al., 2005; Si et al., 2014; Strutt et al., 2014; Liu et al., 2017; Sapotta et al., 2019; Neira et al., 2020) and pillararenes (Cao et al., 2014; Li et al., 2014; Ogoshi et al., 2016; Sathiyajith et al., 2017; Guo H et al., 2020; .Cai et al., 2021). The discovery of “blue box” by Stoddart et al. opened the new era of cationic cyclophanes (Gong et al., 2010; Gong et al., 2011; Dale et al., 2016). Cationic cyclophanes are good candidates for molecular recognition because they not only possess multi-cationic states but also display self-assembly behavior by incorporating *π*-electron-rich guests (Trabolsi et al., 2010a; Barnes et al., 2015; Cheng et al., 2015; Chen et al., 2016; Sun et al., 2017). For example, tetracationic cyclophanes which are constructed on the basis of *π*-electron-deficient 4,4’-bipyridinium units, can selectively complex with *π*-electron-rich guests to form 1:1 or 1:2 complexes (Bühner et al., 1988; Philp et al., 1991; Trabolsi et al., 2010b). Recently, tetraphenylethene (TPE) derivatives which are a classic aggregation-induced emission (AIE) luminophores (Zhou et al., 2018; Ding et al., 2021; Wu H et al., 2021), have been utilized as building blocks to construct macrocyclic compounds (Mei et al., 2015). Attributing to the AIE effect and propeller structure, the TPE-based cyclophanes not only display excellent photophysical properties but also possess flexible and diversified cavity structures which can be explored to capture biomolecules (Luo et al., 2012; Zhao et al., 2012; Kwok et al., 2015). Although a number of TPE-based cationic cyclophanes have been utilized for host–guest recognition, the example of biomolecules recognition in aqueous media by hydrophilic TPE-based cationic cyclophanes is rare.

**SCHEME 1:**
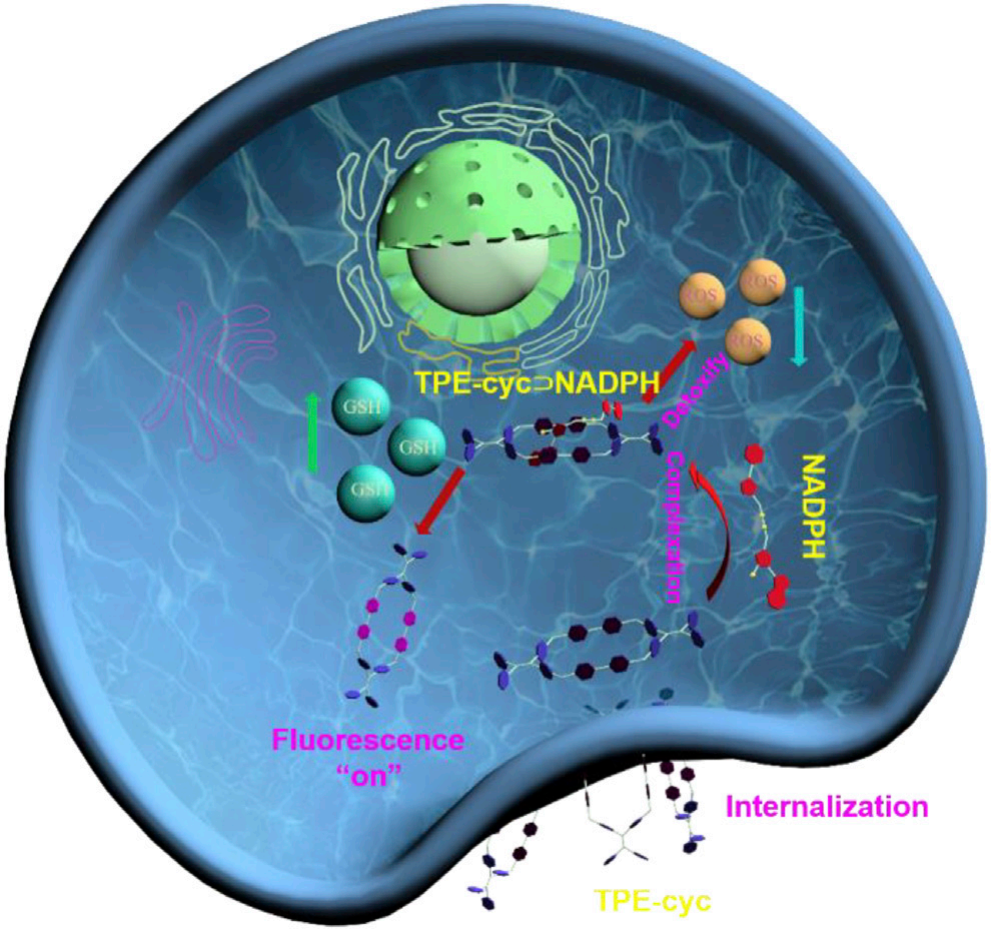
Schematic illustration of the changes of cellular metabolic function regulated by TPE-cyc⊃NADPH.

Here, we synthesized a hydrophilic TPE-based tetracationic cyclophane (TPE-cyc), in which TPE and 4.4’-bipyridinium units acted as building blocks. Attributing to the electrostatic interactions and *π*-*π* stacking, TPE-cyc could specifically recognize NADPH and complex it in a 1:1 manner. After TPE-cyc being internalized by tumor cells, TPE-cyc could capture cellular NADPH to partially break the equilibrium of NADPH-generating reaction (NADH + NADP^+^→NADPH + NAD^+^) and eventually fuel the NADPH-dependent antioxidative ability to detoxify ROS. Meanwhile, the high concentration of GSH in tumor cells could reduce the 4,4’-bipyridinium (MV) units of TPE-cyc into radical cation state and disrupt the photo-induced electron transfer (PET) effect between electron-rich TPE and electron-deficient bipyridinium units, thus recovering the fluorescence of TPE units and lighting up tumor cells (Scheme 1). Hence, TPE-cyc acts as a GSH-responsive fluorescent switch to image cells with high resolution.

## Materials and Methods

### Materials

NADPH was purchased from Sigma. TPE-cyc was synthesized according to literature procedures (Cheng et al., 2019). ^1^H NMR and ^13^C NMR spectra were recorded on a Bruker AvanceⅢ-400 spectrometry. The 2D NOESY NMR spectra were recorded on a Bruker Avance DMX 600 spectrophotometer with TMS as the internal reference. UV-vis-NIR spectra were taken on a Shimadzu UV-3150 spectrophotometer. The fluorescence experiments were measured on an RF-5301 spectrofluorophotometer (Shimadzu Corporation, Japan). The isothermal titration calorimetry (ITC) experiments were performed on a VP-ITC micro-calorimeter (Microcal, United States). The cell images were taken by a confocal laser scanning microscopy (CLSM, Radiance2100, Bio-Rad) with a 100 × oil immersion lens. Flow cytometry measurements were conducted using a FACSCalibur flow cytometer (BD FACSCalibur).

## Methods

### Synthesis of Compound 3

37.1 ml *n*-butyllithium (1.6 M) was added into a solution of 1 (10 g, 59.4 mmol) in dry THF (100 ml) at 0°C under N_2_ atmosphere. The orange-red solution was stirred for 0.5 h at 0°C. Then, 2 (6.3 g, 29.7 mmol) was dissolved in dry THF and dropwise added to the above mixture. The resulting solution was allowed to warm to room temperature and still stirred for 8 h. At last, saturated ammonium chloride solution was added to quench the reaction. The mixture was extracted with DCM three times. The organic phase were combined and dried over anhydrous Na_2_SO_4_. The solvent was removed by rotary evaporation and the acquired crude product was dissolved in toluene with the 4Å molecular sieve dehydration unit. After addition of catalytic *p*-toluenesulphonic acid (342 mg, 1.8 mmol), the toluene solution was refluxed for 5 h and the generated H_2_O was separated. The organic layer was washed with 10% NaHCO_3_ aqueous solution and dried over anhydrous Na_2_SO_4_. After removal of toluene, the obtained mixture was purified by column chromatography (silica gel; petroleum ether) to obtain 3 as a white solid (9.0 g, 85%).

### Synthesis of Compound 4

Under a N_2_ atmosphere, 3 (1.1 g, 2.83 mmol) was first dissolved in CCl_4_ (20 ml), then dibenzoyl peroxide (50 mg, 0.2 mmol) and NBS (1.5 g, 8.49 mmol) were added. The mixture was heated to reflux for 12 h. After reaction, the solution was filtered to remove solid impurity and the CCl_4_ solution was washed with brine three times. The organic phase were collected and dried by anhydrous Na_2_SO_4_. The CCl_4_ was evaporated and the crude product was purified by a silica gel column chromatography (silica gel; petroleum ether) to obtain 4 as a white solid (0.9 g, 60%).

### Synthesis of Compound 5

4,4’-bipyridine (2.0 g, 12.8 mmol) was dissolved in CH_3_CN (20 ml)and was heated to reflux. Next, compound 4 (1.2 mg, 2.3 mmol) was dissolved in CH_3_CN (5 ml) and dropwise added to the bipyridine solution. The resulting mixture was refluxed for 3 days. The formed precipitate was filtered and washed with CH_3_CN three times, and compound 5 was obtained after dry under high vacuum (1.7 g, 90%).

### Synthesis of Compound 6

Tetrabutylammonium iodide (TBAI, 35 mg, 0.095 mmol), 4 (220 mg, 0.42 mmol) and 5 (400 mg, 0.42 mmol) were dissolved in dry CH_3_CN (100 ml) and was heated at 85°C for 72 h. After reaction, the orange product was acquired by centrifuge and dried under high vacuum. After NH_4_PF_6_ anion conversion in water, six was obtained as a pale yellow solid (250 mg, 37%).

### Synthesis of Compound TPE-Cyc

Tetrabutylammonium chloride (TBACl, 205.66 mg, 0.74 mmol) and 6 (100 mg, 0.074 mmol) were dissolved in CH_3_CN and stirred overnight. After reaction, the orange terreous product was acquired by centrifuge and washed with CH_3_CN three times. TPE-cyc was obtained after dry in high vacuum (61 mg, 90%).

## Results and Discussion

### Investigation of Host–Guest Complexation Between TPE-Cyc and NADPH

**FIGURE 1:**
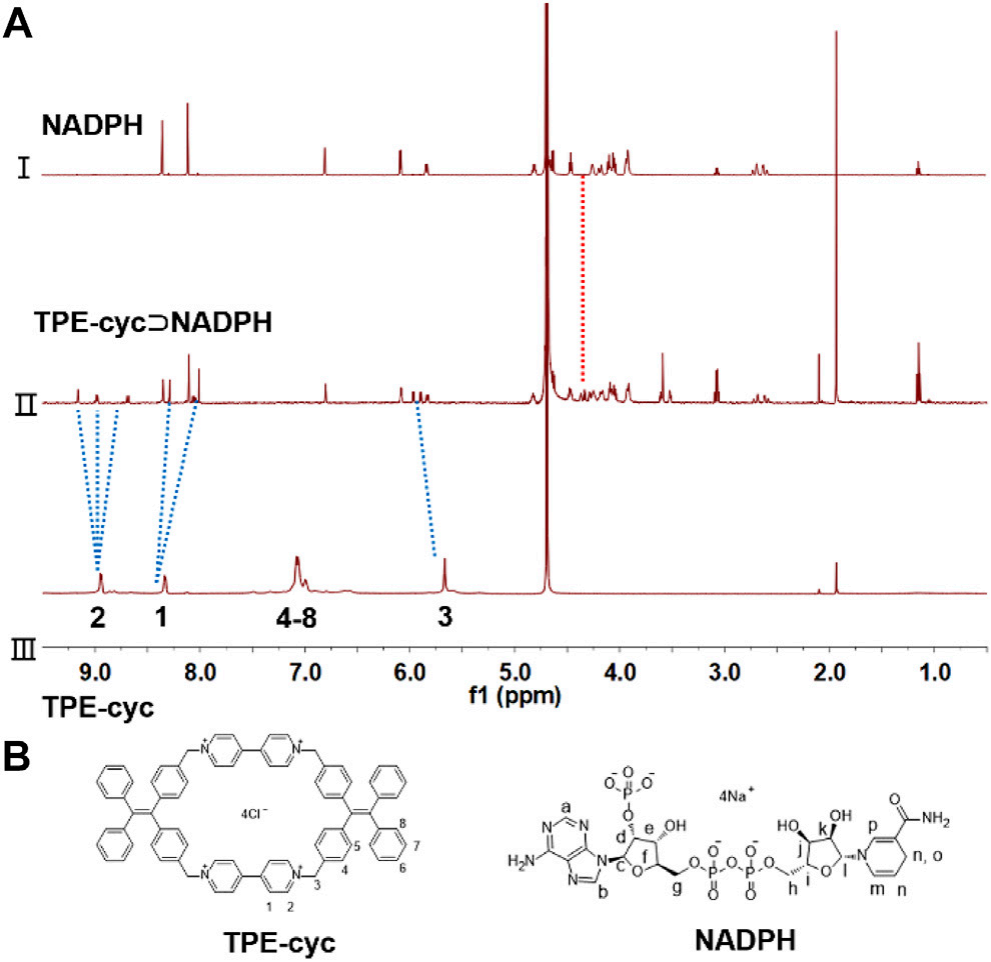
**(A)** Partial ^1^H NMR spectra (D_2_O, room temperature, 400 MHz): (Ⅰ) NADPH (1.00 mM). (Ⅱ) TPE-cyc⊃NADPH [TPE-cyc (1.00 mM) and NADPH (1.00 mM)]. (Ⅲ) TPE-cyc (1.00 mM). **(B)** Chemical structures of TPE-cyc and NADPH.

**FIGURE 2:**
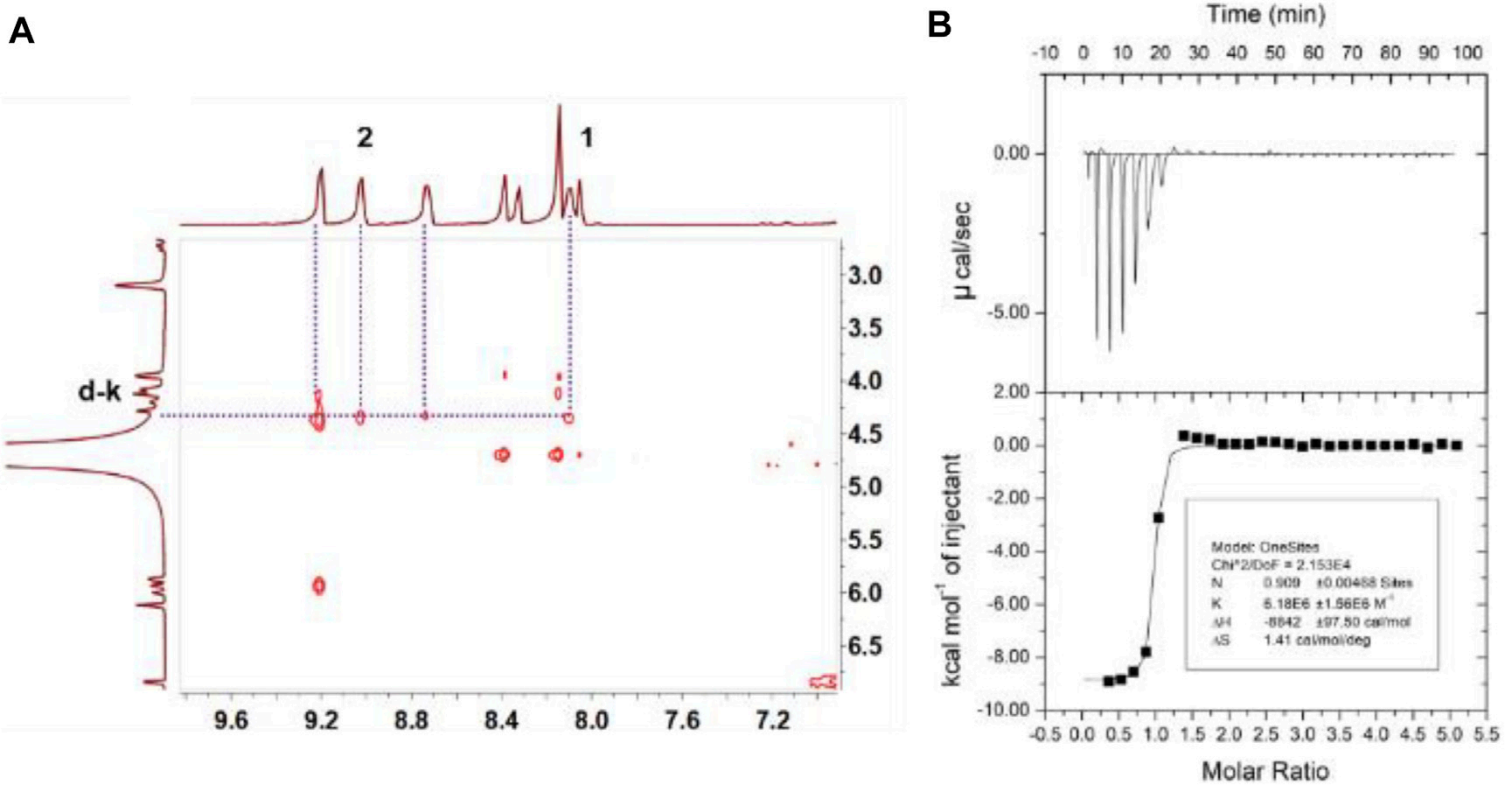
**(A)** Partial NOESY NMR spectra (600 MHz, D_2_O, room temperature) of TPE-cyc (1.0 mM) and NADPH (1.0 mM). **(B)** Microcalorimetric titration of TPE-cyc (2.00 mM, 10 µl per injection) with NADPH (0.100 mM) in water at 298.15 K.

TPE-cyc was synthesized *via* a two-step S_N_2 reaction as shown in Scheme 1. To investigate the host–guest interaction between TPE-cyc and NADPH, ^1^H NMR spectroscopy was conducted in D_2_O. As shown in Figures 1A,B, when an equimolar amount of NADPH and TPE-cyc were mixed in D2O, apparent chemical shift changes of the protons on TPE-cyc were observed. For instance, the signals of protons H_1_, H_2_ and H_3_ on TPE-cyc were divided into multiple sets of sharp signals with obvious chemical shift changes, which may be induced by the electrostatic interaction between the pyridinium unit and tetraphosphate unit of NADPH. Meanwhile, the signals of protons H_4-8_ disappeared completely after complexation. All these results provided compelling evidence for the host–guest interactions between TPE-cyc and NADPH (Cheng et al., 2019). In addition, the signals of protons on NADPH also displayed obvious peak broadening effect, further indicating the occurrence of host–guest complexation. On the other hand, there were H_1_-H_d-k_ and H_2_-H_d-k_ inter-correlation between TPE-cyc and NADPH in nuclear overhauser effect spectroscopy (NOESY) spectrum (Figure 2A), confirming the formation of TPE-cyc⊃NADPH.

**FIGURE 3:**
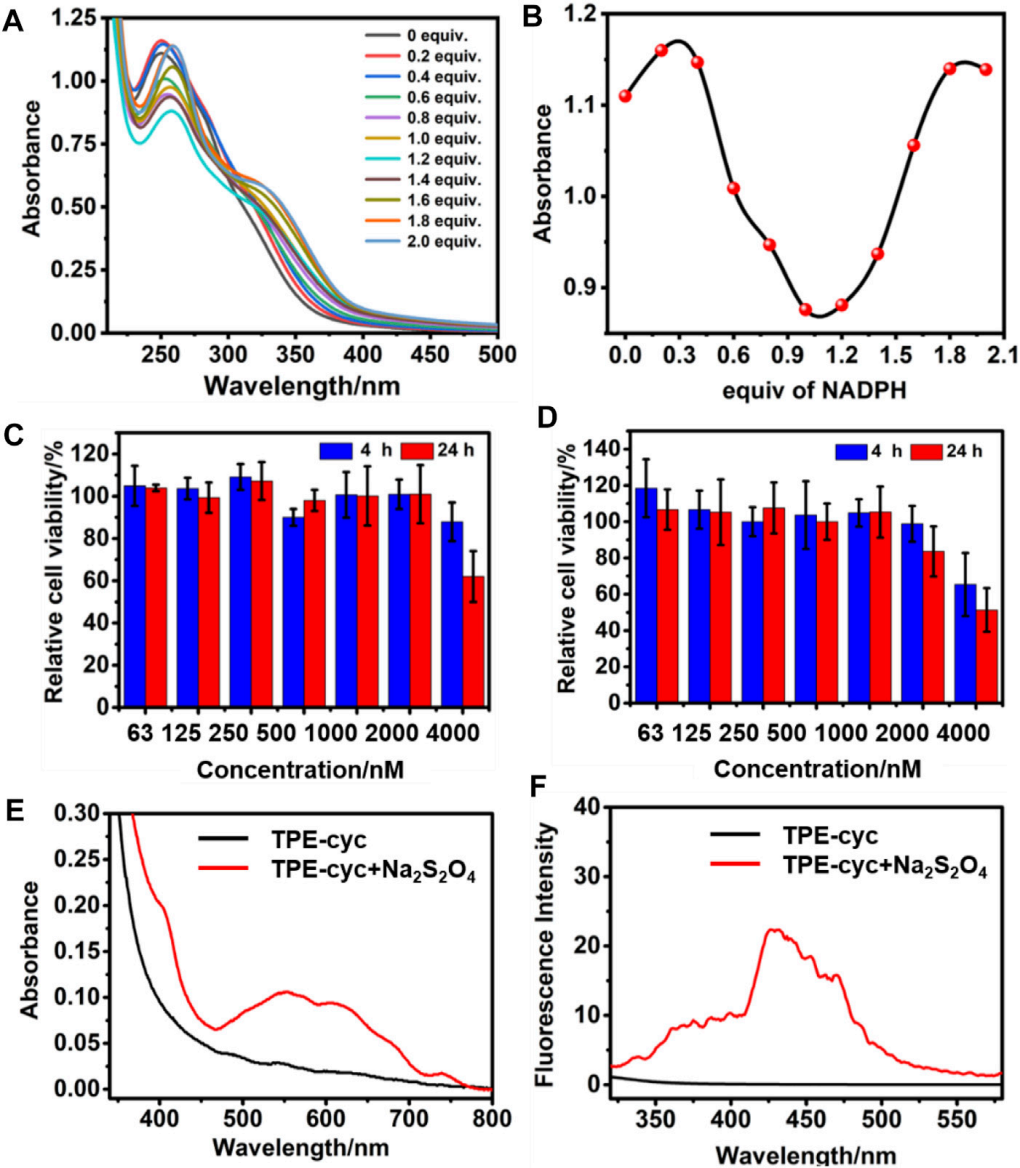
**(A)** UV/vis spectra of TPE-cyc in the presence of different amounts of NADPH. **(B)** The plots of UV absorption maximum of TPE-cyc in the presence of different amounts of NADPH. Cytotoxicity of **(C)** HeLa and **(D)** U87 cells incubated with different concentrations of TPE-cyc for 4 and 24 h **(E)** UV/vis spectra of TPE-cyc with or without Na_2_S_2_O_4_. **(F)** Fluorescence spectra of TPE-cyc with or without Na_2_S_2_O_4_.

Isothermal titration calorimetry (ITC) experiment was carried to provide the thermodynamic energy information for the complexation. As shown in Figure 2B, the *K*_a_ value of TPE-cyc⊃NADPH was calculated to be (6.18 ± 1.56) × 10^6^ M^−1^ and the stoichiometry was 1:1. The acquired information of entropy and enthalpy changes (ΔH° < 0; TΔS° > 0) from ITC data demonstrated that this supramolecular complexation was promoted by a beneficial entropy-assisted enthalpy change. The driving forces of the molecular recognition were the synergistic effect of hydrophobic interaction, electrostatic interaction and *π*-*π* stacking interaction. Furthermore, there was a fragment peak *m/z* = 948.3 [corresponding to (TPE-cyc‧NADPH-3Cl-H)^2+^] in electrospray ionization mass spectrometry (Supplementary Figure S9), which further demonstrated the formation of a 1:1 TPE-cyc⊃NADPH complex. The UV-vis absorption was also conducted to investigate the complexation between TPE-cyc and NADPH. As shown in Figure 3A, by the continual addition of NADPH into TPE-cyc solution, the maximum absorbance of TPE-cyc at 250 nm gradually decreased and moved to 258 nm, and the maximum decrease occurred when 1.0 equiv. of NADPH was added (Figure 3B), supporting a 1:1 stoichiometry of TPE-cyc⊃NADPH.

### Investigation of Cell Viability Regulated by TPE-Cyc

NADPH is known as a crucial co-enzyme in the event of cellular electron transfer which drives the biosynthesis of amino acids, DNA, phospholipids, fatty acids and steroids. Another important function of NADPH originating from the powerful reducibility of NADPH is to fuel the activities of various enzymes, such as glutathione peroxidase (GSHPx), catalase and superoxide dismutase, which play an important role in permitting microorganisms to survive in aerobic environment. Hence, breaking the balance of NADPH in living system can induce severe damage for cells, even death. Considering the strong complexing ability of TPE-cyc for NADPH, we investigate the impact of TPE-cyc on biological functions of cells.

The cell viability was firstly assessed by a 3-(4′,5′-dimethylthiazol-2′-yl)-2,5-diphenyl tetrazolium bromide (MTT) assay, wherein U87 and HeLa cells were incubated with different concentrations of TPE-cyc for 4 or 24 h. As shown in Figures 3C,D, cell viability maintained nearly 100% survival rate in the range of 0.063–1.0 μM, suggesting this concentration range of TPE-cyc could not disrupt the NADPH balance. However, the survival rate decreased with the further increase of concentration. For example, when the concentration reached 4 μM, the survival rate fell by half (Figures 3C,D), indicating this concentration of TPE-cyc could capture enough intracellular NADPH and eventually induce cell death. In addition, the cytotoxicity of six was similar to TPE-cyc, suggesting tetracationic macrocyclic structure was the key of cytotoxicity against cells (Supplementary Figure S11).

### Investigation of Antioxidative Ability of Tumor Cells Regulated by TPE-Cyc

**FIGURE 4:**
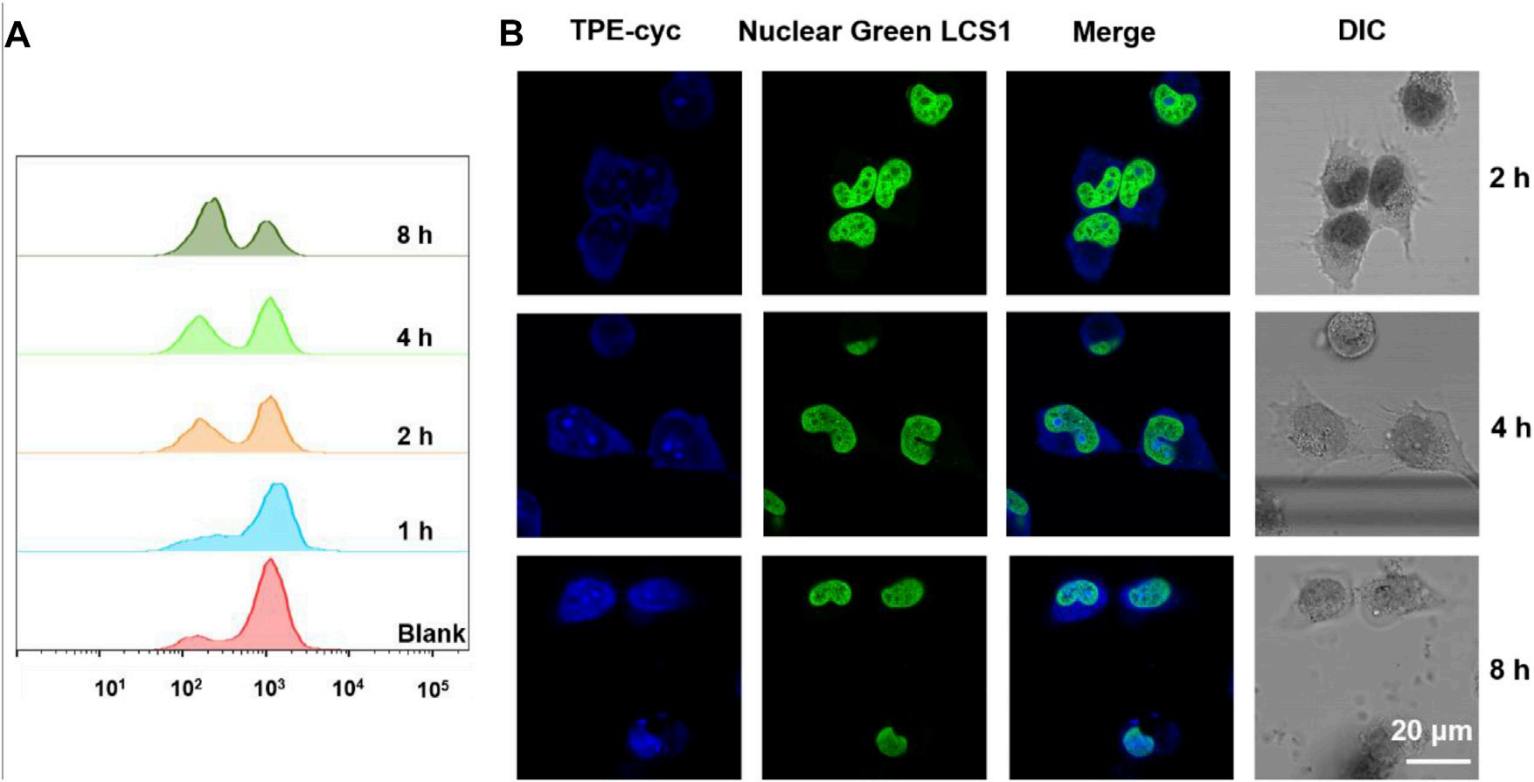
**(A)** Flow cytometry results of ROS fluorescence signals in HeLa cells incubated with TPE-cyc for different time periods. **(B)** Confocal images of HeLa cells incubated with TPE-cyc for different time periods (Nuclear Green LCS1 was used to stain nuclei of living HeLa cells).

NADPH has an important ability to keep glutathione in its reduction form GSH, which eliminates ROS and transforms harmful hydrogen peroxide into water under the help of GSHPx (Margis et al., 2008). Therefore, NADPH plays a vital role in resisting cellular oxidative stress. We utilized 2′,7′-dichlorofluorescin diacetate (DCF-DA) as a fluorescence probe to monitor the intracellular ROS level (Li et al., 2018; Ren et al., 2020). DCFH-DA itself does not emit fluorescence, but the intracellular ROS can oxidize the non-emissive DCF-DA into emissive DCF. As shown in Supplementary Figure S12, with the increase of incubation time, the level of ROS in TPE-cyc group decreased, suggesting the antioxidative ability of cells was enhanced. Flow cytometry (FCM) was also applied to quantitatively analyze the level of intracellular ROS. Similar to the result of fluorescence imaging, ROS production significantly decreased with the extension of time (Figure 4A). The reason may be ascribed that the capture of intracellular NADPH by TPE-cyc broke the equilibrium of NADPH-involving redox process and triggered the generation of reducing substances which can detoxify ROS.

### Investigation of Cell Imaging Ability of TPE-Cyc

Owing to the PET effect between TPE and bipyridinium units, water-soluble TPE-cyc is a quencher-type host and it has an intense UV absorption but no fluorescence (Hayashida and Kaku, 2013), which severely limits its expansion and application. In the reductive condition, MV^2+^ can be reduced into MV^⋅+^ and the PET effect between TPE and bipyridinium units is disrupted (Jiao et al., 2019; Guo Q. H et al., 2020; Zhang et al., 2020), which may be used to initiate the fluorescence of TPE-cyc. As shown in Figure 3E, there was a characteristic peak of MV^⋅+^ in the range of 450–750 nm after addition of Na_2_S_2_O_4_, indicating MV^2+^ was reduced into MV^⋅+^ with the help of reducing agent (Lomoth et al., 2002). As expected, there was a new fluorescence emission within the range of 300–550 nm in the TPE-cyc + Na2S_2_O_4_ group, suggesting the fluorescence of TPE-cyc was initiated. In tumor cells, the concentration of GSH is high, which can be utilized to light up TPE-cyc. Confocal laser scanning microscopy (CLSM) was utilized to study the internalization behavior of TPE-cyc. As shown in Figure 4B, after 2 h incubation, apparent blue fluorescence arising from TPE-cyc was observed in the cytoplasm, indicating that TPE-cyc was easily internalized by HeLa cells. With the incubation time increased to 8 h, the intensity of blue fluorescence significantly increased, suggesting the endocytosis of TPE-cyc occurred in a time-dependent mode and intracellular reducing environment ensured continuous luminous of TPE-cyc, which provided a crucial advantage for TPE-cyc to be applied in fluorescence imaging.

## Conclusion

In summary, a hydrophilic tetracationic cyclophane was constructed based on TPE and 4,4’-bipyridinium units. Attributing to the large and flat rectangle-like cavity, TPE-cyc could specifically recognize NADPH and form a 1:1 host–guest complex. TPE-cyc was not only easily phagocytized by tumor cells but also able to selectively capture cellular NADPH, thus breaking the equilibrium of NADPH-involving redox process and improving the antioxidative ability to eliminate ROS. Meanwhile, the sufficient intracellular reducing environment such as the high concentration of GSH reduced MV2+ unit of TPE-cyc into MV^⋅+^ to forbid the PET effect between TPE and MV units, realizing the fluorescence recovery of TPE-cyc and eventually fluorescence imaging of tumor cells with high resolution. This current study opens a door for cationic cyclophanes to broaden their biological applications in recognizing biomacromolecules and imaging tumor cells, which has a great potential to diagnose and treat difficult miscellaneous diseases of humans in the future.

## Data Availability

The original contributions presented in the study are included in the article/Supplementary Material, further inquiries can be directed to the corresponding authors.
