# Supplementary material for: Hydrophilic Tetraphenylethene-Based Tetracationic Cyclophanes: NADPH Recognition and Cell Imaging With Fluorescent Switch
Source: Front Chem. 2021 Dec 22;9:817720. doi: 10.3389/fchem.2021.817720 (PMC8727463; doi:10.3389/fchem.2021.817720)
Supplement: Supplementary file 1 [file DataSheet1.docx]

**Hydrophilic tetraphenylethene-based tetracationic cyclophanes: NADPH recognition and cell imaging with fluorescent switch**†

Dan Wu,^1,^* Zhankui Zhang,^1,^ Xinyang Yu,^2^ Bing Bai^2^ and Shaolong Qi^3,^*

*^1^ College of Materials Science and Engineering, Zhejiang University of Technology, Hangzhou, 310014, P. R. China; Email address:* [*danwu@*](mailto:danwu@)*zjut.edu.cn*

*^2^* *Key Laboratory of Bioorganic Phosphorus Chemistry & Chemical Biology, Department of Chemistry, Tsinghua University, Beijing 100084, P. R. China*

*^3^ Key Laboratory & Engineering Laboratory of Lymphatic Surgery Jilin Province, China-Japan Union Hospital of Jilin University, Changchun, 130031, P. R. China; Email address:* *15584187168@163.com*

**Electronic Supplementary Information (14 pages)**

| 1. | *Materials and methods* | S2 |
| --- | --- | --- |
| 2. | *The synthesis of* ***TPE-cyc*** | S3 |
| 3. | *ESI-MS spectrum of* ***TPE-cyc⊃NADPH*** *in H_2_O* | S9 |
| 4. | *Job’s plots obtained by recording the absorption maximum of* ***TPE-cyc*** *and NADPH* | S10 |
| 5. | *Cytotoxicity evaluation* | S10 |
| 6. | *Fluorescent images of ROS in HeLa cells incubated with* ***TPE-cyc*** | S11 |

1. Materials and methods

All reagents were commercially available and used as supplied without further purification. Solvents were either employed as purchased or dried according to procedures described in the literature. Nicotinamide adenine dinucleotide phosphate (NADPH) was purchased from Sigma. **TPE-cyc** was synthesized according to literature procedures.^S1 1^H NMR and ^13^C NMR spectra were recorded on a Bruker Avance Ⅲ-400 spectrometry. The 2D NOESY NMR spectra were recorded on a Bruker Avance DMX 600 spectrophotometer with TMS as the internal reference. Transmission electron microscopy (TEM) investigations were carried out on a HT-7700 instrument. UV-vis-NIR spectra were taken on a Shimadzu UV-3150 spectrophotometer. The fluorescence experiments were measured on an RF-5301 spectrofluorophotometer (Shimadzu Corporation, Japan). The isothermal titration calorimetry (ITC) experiments were performed on a VP-ITC micro-calorimeter (Microcal, USA). The cell images were taken by a confocal laser scanning microscopy (CLSM, Radiance2100, Bio-Rad) with a 100 × oil immersion lens. Flow cytometry measurements were conducted using a FACSCalibur flow cytometer (BD FACSCalibur).

2. The synthesis of **TPE-cyc**


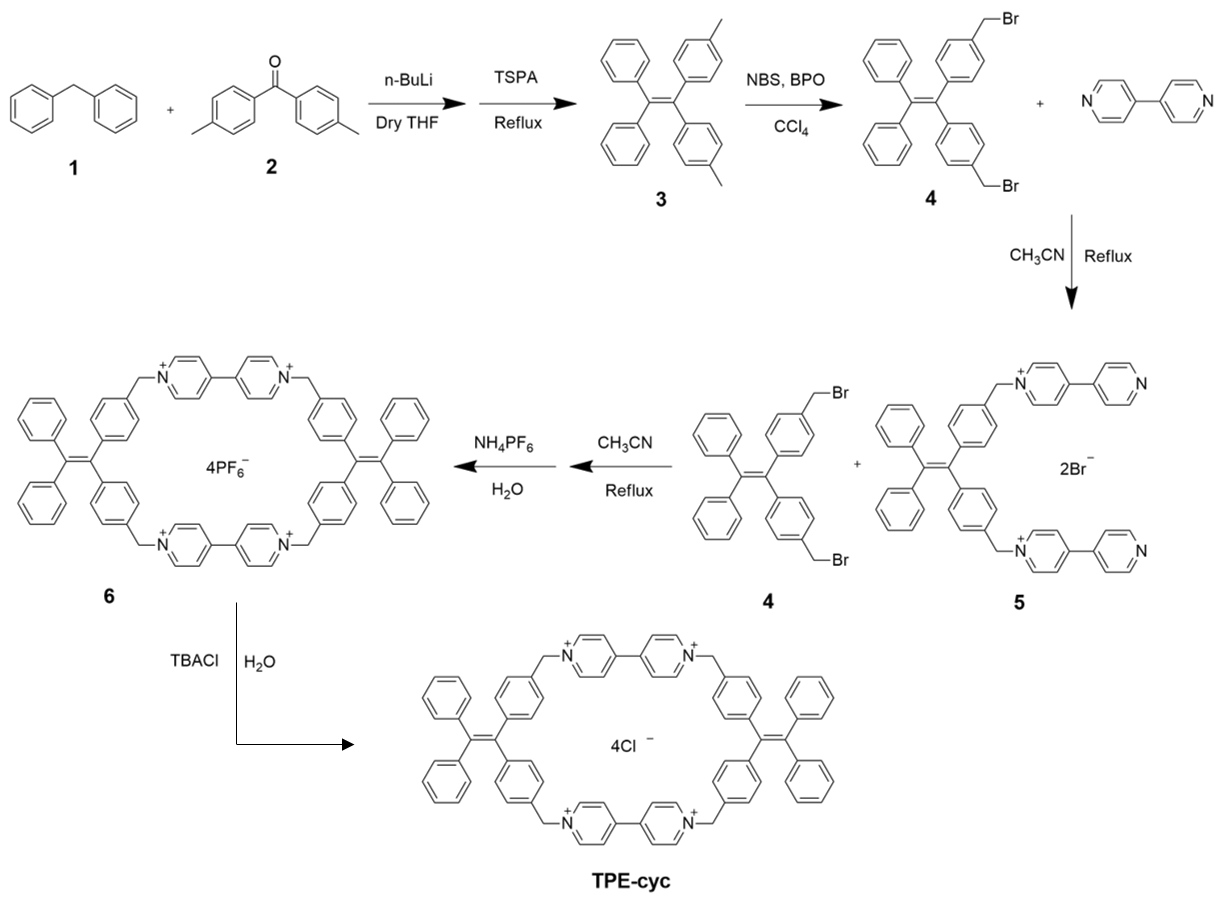


***Scheme S1.*** Synthetic route to **TPE-cyc**.

Synthesis of **3**: 37.1 mL *n*-butyllithium (1.6 M) was added into a solution of **1** (10 g, 59.4 mmol) in dry THF (100 mL) at 0 ℃ under a N_2_ atmosphere. The resulting orange-red solution was stirred for 30 min at 0 ℃. Next, **2** (6.3 g, 29.7 mmol) was dissolved in dry THF (20 mL). This solution was dropwise added to the above mixture, and the mixture was allowed to warm to room temperature and still stirred for 6 h. The reaction was quenched by an aqueous solution of saturated ammonium chloride. The organic phase was collected and the aqueous phase was extracted with DCM three times. The organic phase were combined and dried over anhydrous Na_2_SO_4_. The solvent was evaporated and the resulting crude alcohol was dissolved in toluene with the 4Å molecular sieve dehydration unit. A catalytic amount of *p*-toluenesulphonic acid (342 mg, 1.8 mmol) was added and the mixture was refluxed for 5 h and the generated H_2_O was separated. The toluene layer was washed with 10% aqueous NaHCO_3_ solution and dried over anhydrous Na_2_SO_4_. Upon removal of toluene by rotary evaporation, the obtained mixture was further purified by column chromatography (silica gel; petroleum ether) to obtain **3** as a white solid (9.0 g, 85%). The ^1^H NMR spectrum of **3** is shown in Fig. S1. ^1^H NMR (400 MHz, CDCl_3_, room temperature) *δ* (ppm): 7.14−7.10 (m, 6H), 7.06−7.04 (m, 4H), 6.95−6.91 (m, 8H), 2.28 (s, 6H).


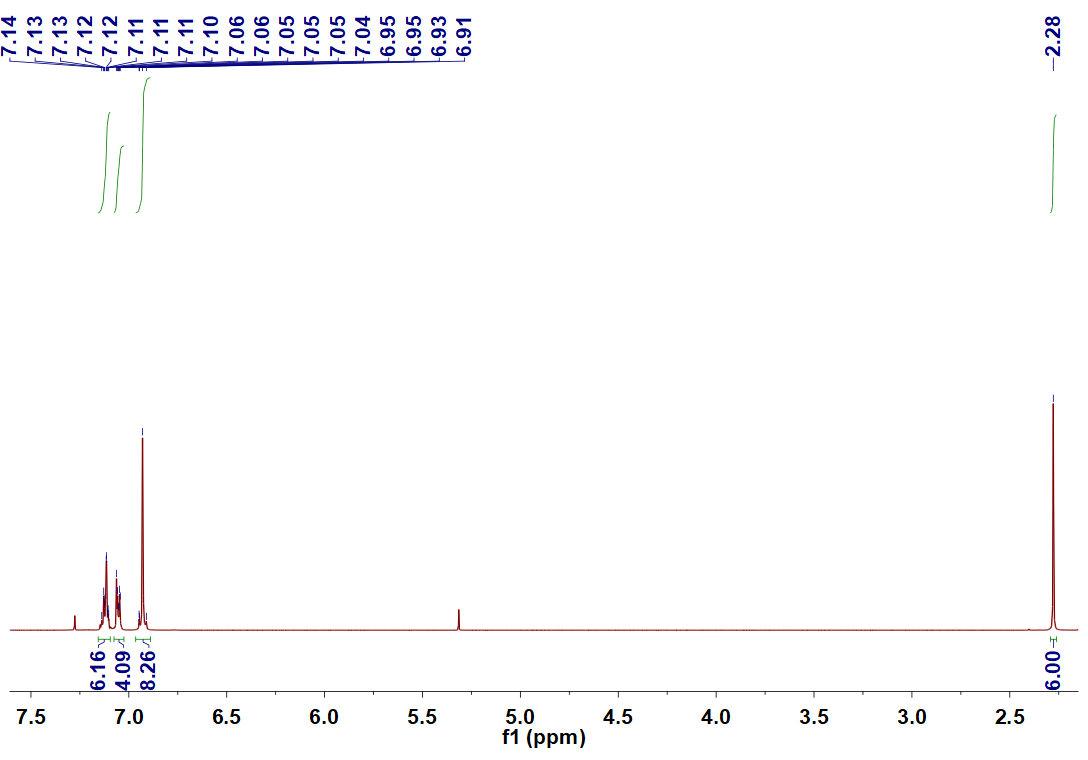


***Fig. S1*** ^1^H NMR spectrum (400 MHz, CDCl_3_, room temperature) of **3**.

Synthesis of **4**: Under a N2 atmosphere, **3** (1.1 g, 2.83 mmol) was first dissolved in CCl4 (20 mL), then NBS (1.5 g, 8.49 mmol) and dibenzoyl peroxide (50 mg, 0.2 mmol) were added to the solution. The mixture was heated to reflux for another 12 h. After reaction, the mixture was filtered to remove solid impurity. Next, DCM was added to the filtrate and the solution was washed with brine three times. The organic phase were combined and dried over anhydrous Na_2_SO_4_. The solvent was evaporated and the residue was purified by a silica gel column chromatography (silica gel; petroleum ether) to obtain **4** as a white solid (0.9 g, 60%). The ^1^H NMR spectrum of **4** is shown in Fig. S2. ^1^H NMR (400 MHz, CDCl_3_, room temperature) *δ* (ppm): 7.15−7.12 (m, 10H), 7.03−6.99 (m, 8H), 4.43 (s, 4H). The ^13^C NMR spectrum of **4** is shown in Fig. S3. ^13^C NMR (100 MHz, CDCl_3_, room temperature) *δ* (ppm): 146.4, 143.9, 133.0, 132.9, 131.8, 131.7, 131.6, 129.9, 129.6, 128.9, 128.8, 128.4, 128.3, 127.9, 65.3, 65.2.


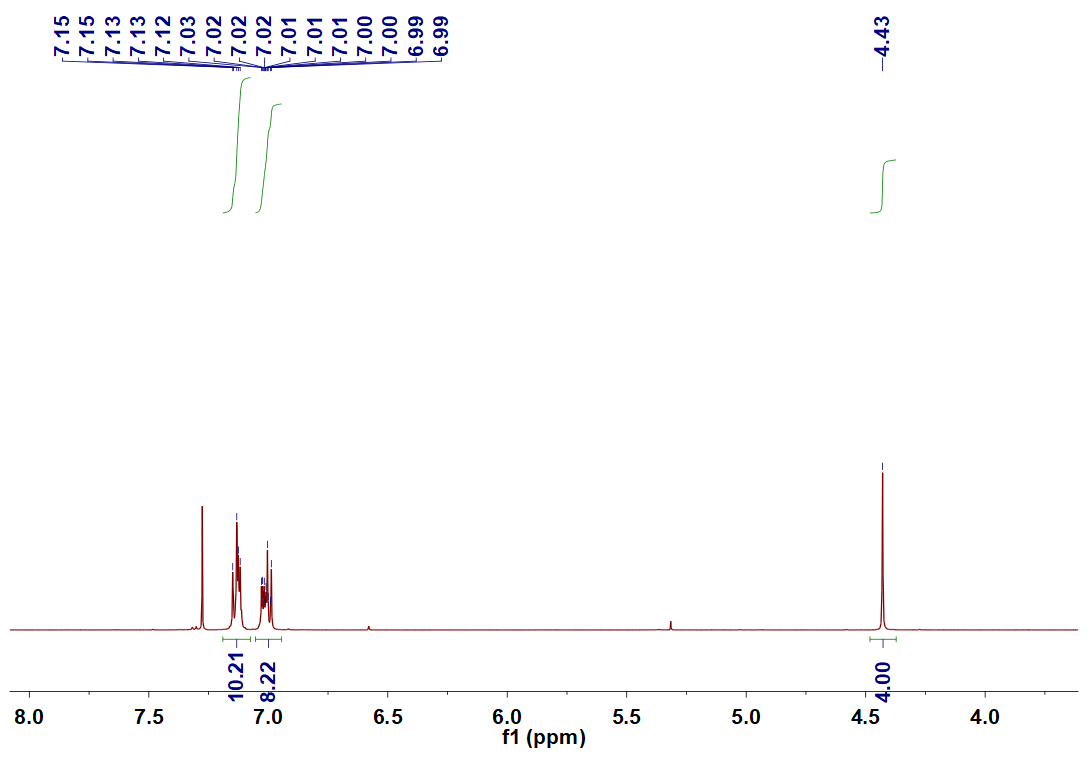


***Fig. S2*** ^1^H NMR spectrum (400 MHz, CDCl_3_, room temperature) of **4**.


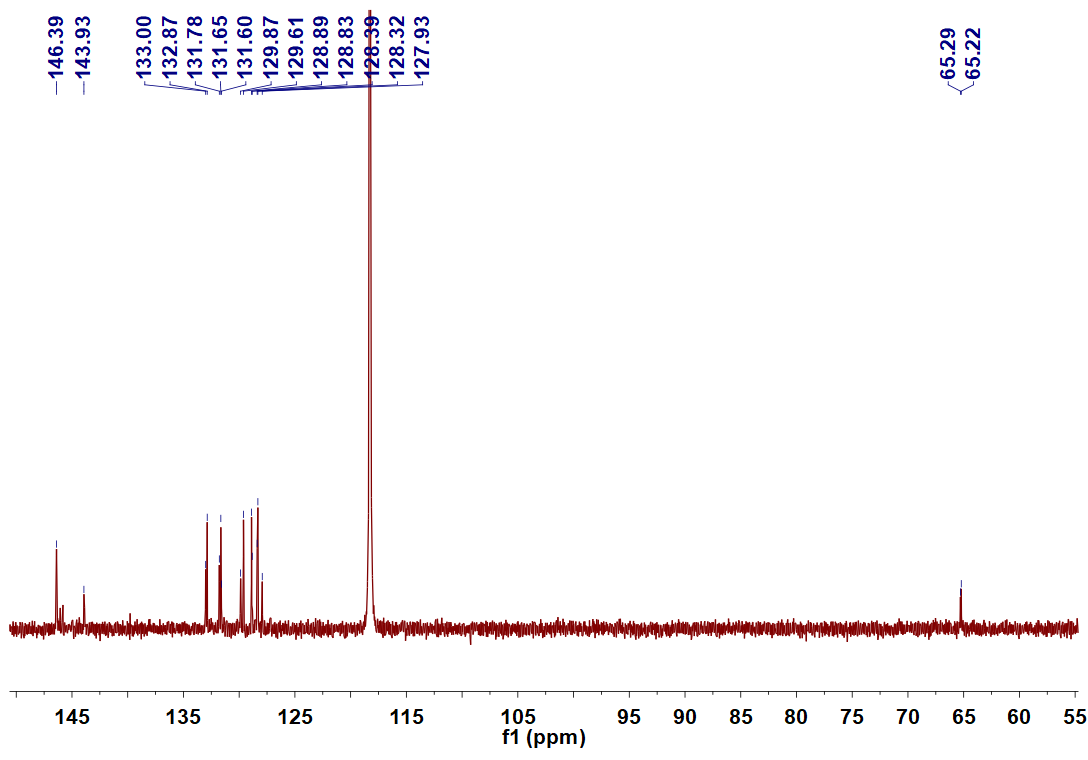


***Fig. S3*** ^13^C NMR spectrum (100 MHz, CDCl_3_, room temperature) of **4**.

Synthesis of **5**: 4,4’-bipyridine (2.0 g, 12.8 mmol) was dissolved in 20 mL of CH3CN and the solution was heated to reflux. Next, compound **4** (1.2 mg, 2.3 mmol) was dissolved in CH3CN (5 mL) and was dropwise added to the bipyridine solution. The mixture was refluxed for 3 days. The formed precipitate was filtered and washed with acetonitrile three times, and compound **5** was obtained after dry under high vacuum (1.7 g, 90%). The ^1^H NMR spectrum of **5** is shown in Fig. S4. ^1^H NMR (400 MHz, CDCl_3_, room temperature) *δ* (ppm): 8.88−8.87 (m, 4H), 8.76−8.75 (m, 4H), 8.34−8.32 (m, 4H), 7.82−7.80 (m, 4H), 7.26−7.24 (m, 4H), 7.18−7.16 (m, 10H), 7.05−7.03 (m, 4H), 5.67 (s, 4H). The ^13^C NMR spectrum of **5** is shown in Fig. S5. ^13^C NMR (100 MHz, CDCl_3_, room temperature) *δ* (ppm): 155.6, 152.1, 145.9, 145.7, 143.9, 142.0, 132.9, 132.0, 131.8, 129.6, 128.8, 127.9, 127.1, 122.8, 64.7.


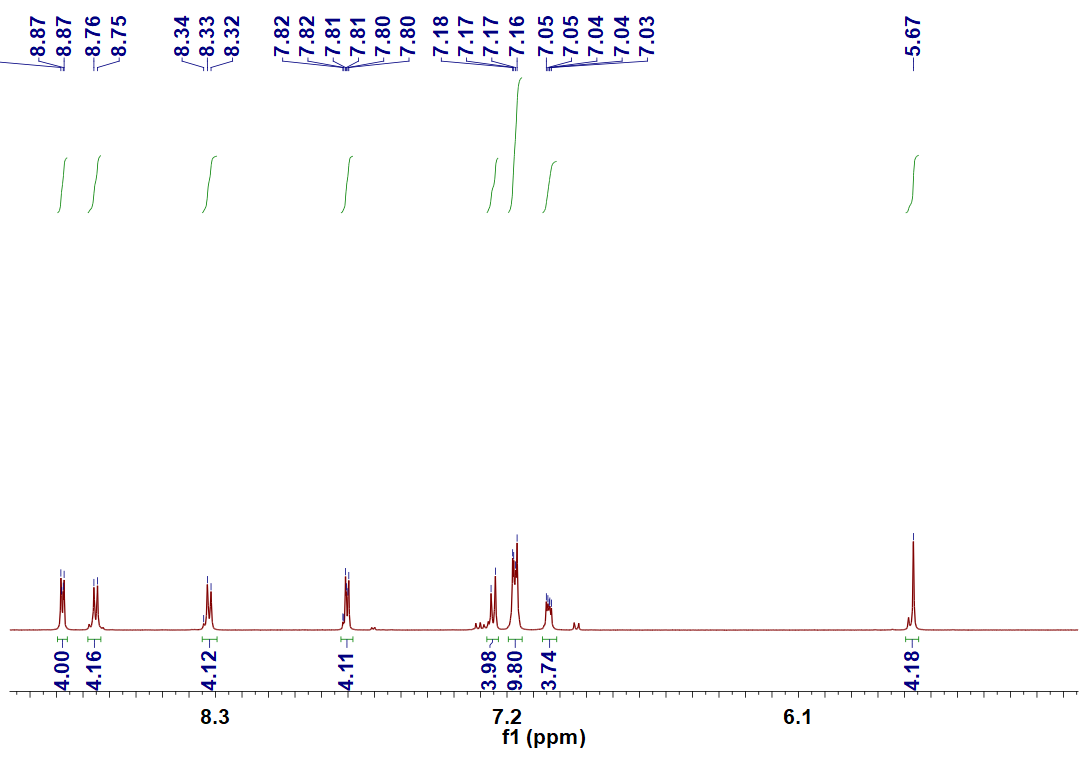


***Fig. S4*** ^1^H NMR spectrum (400 MHz, CDCl_3_, room temperature) of **5**.


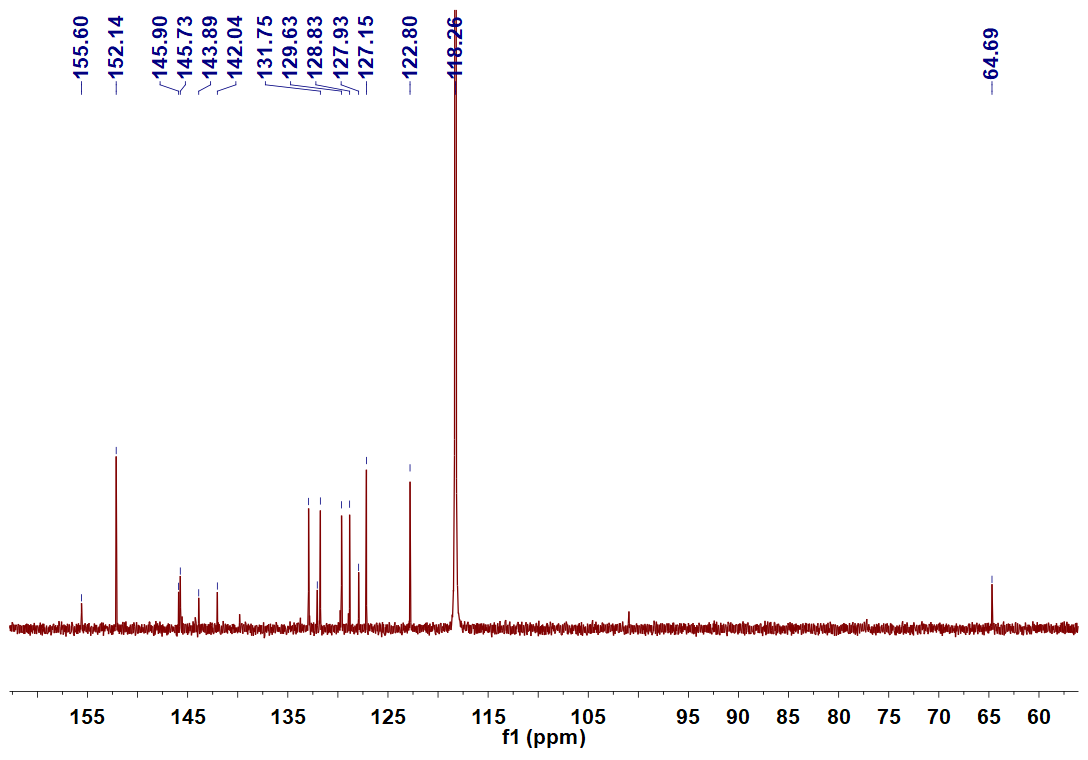


***Fig. S5*** ^13^C NMR spectrum (100 MHz, CDCl_3_, room temperature) of **5**.

Synthesis of **6**: Compound **4** (220 mg, 0.42 mmol), compound **5** (400 mg, 0.42 mmol), and tetrabutylammonium iodide (TBAI, 35 mg, 0.095 mmol) were dissolved in dry CH_3_CN (100 mL) and was heated at 85 °C for 72 h. Then the orange crude product was obtained by centrifuge and was dried under high vacuum. After anion conversion in water by addition of an excess of NH_4_PF_6_, **6** was obtained as a pale yellow solid (250 mg, 37%). The ^1^H NMR spectrum of **6** is shown in Fig. S6. ^1^H NMR (400 MHz, CDCl_3_, room temperature) *δ* (ppm): 9.43−9.39 (m, 8H), 8.95−8.84 (m, 8H), 7.81−7.70 (m, 28H), 7.63−7.57 (m, 8H). The ^13^C NMR spectrum of **6** is shown in Fig. S7. ^13^C NMR (100 MHz, CDCl_3_, room temperature) *δ* (ppm): 151.3, 151.1, 146.4, 146.0, 145.8, 143.9, 139.8, 133.0, 132.9, 131.8, 131.7, 131.6, 129.9, 129.6, 128.9, 129.6, 128.9, 128.8, 128.3, 127.9, 65.3, 65.2.


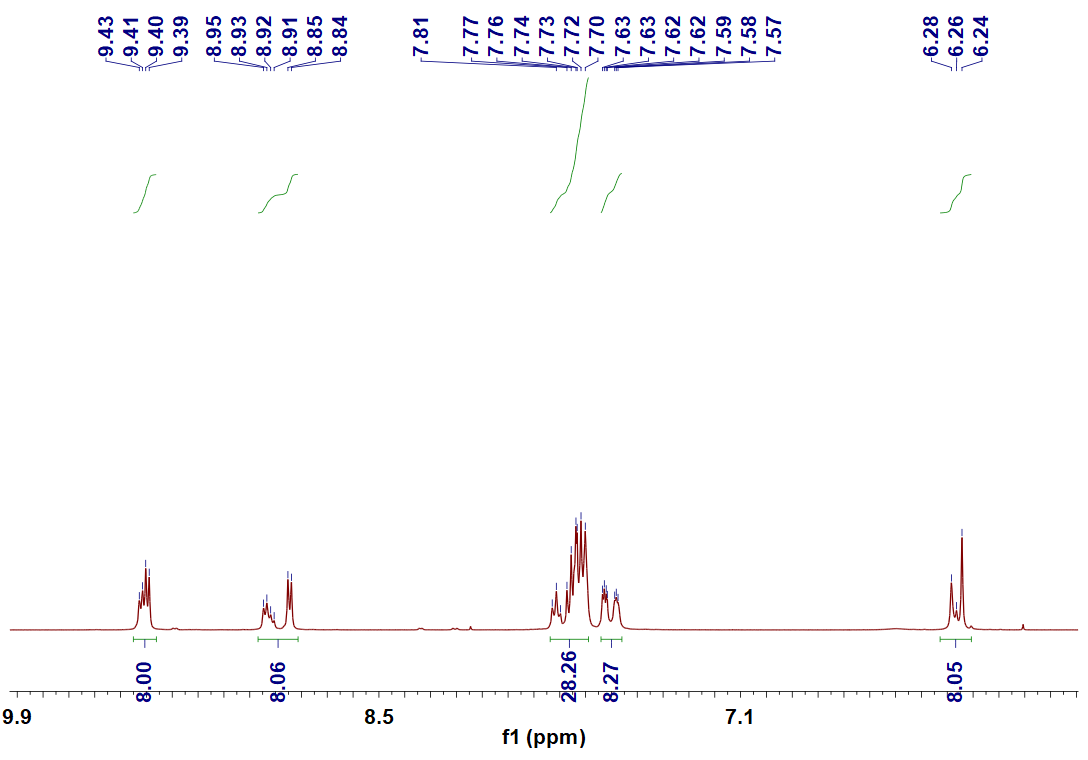


***Fig. S6*** ^1^H NMR spectrum (400 MHz, CDCl_3_, room temperature) of **7**.


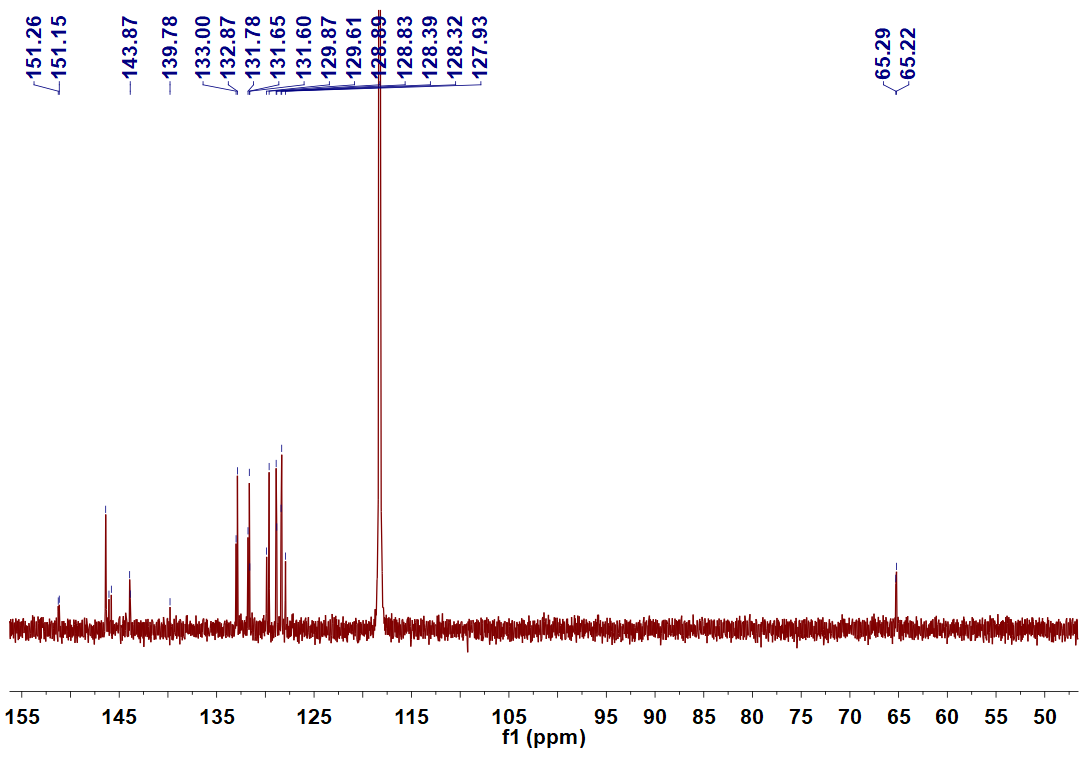


***Fig. S7*** ^13^C NMR spectrum (100 MHz, CDCl_3_, room temperature) of **6**.

Synthesis of **TPE-cyc**: An excess of TBACl (205.66 mg, 0.74mmol) was added to the acetonitrile solution of **6** (100 mg, 0.074 mmol) and stirred overnight at room temperature. Then the mixture was centrifuged and washed with acetonitrile three times. **TPE-cyc** was obtained after dry in high vacuum (61 mg, 90%). The ^1^H NMR spectrum of **TPE-cyc** is shown in Fig. S8. ^1^H NMR (400 MHz, D_2_O, room temperature) *δ* (ppm): 8.99−8.98 (m, 8H), 8.38−8.37 (m, 8H), 7.11−6.97 (m, 36H), 6.97−6.96 (m, 8H).


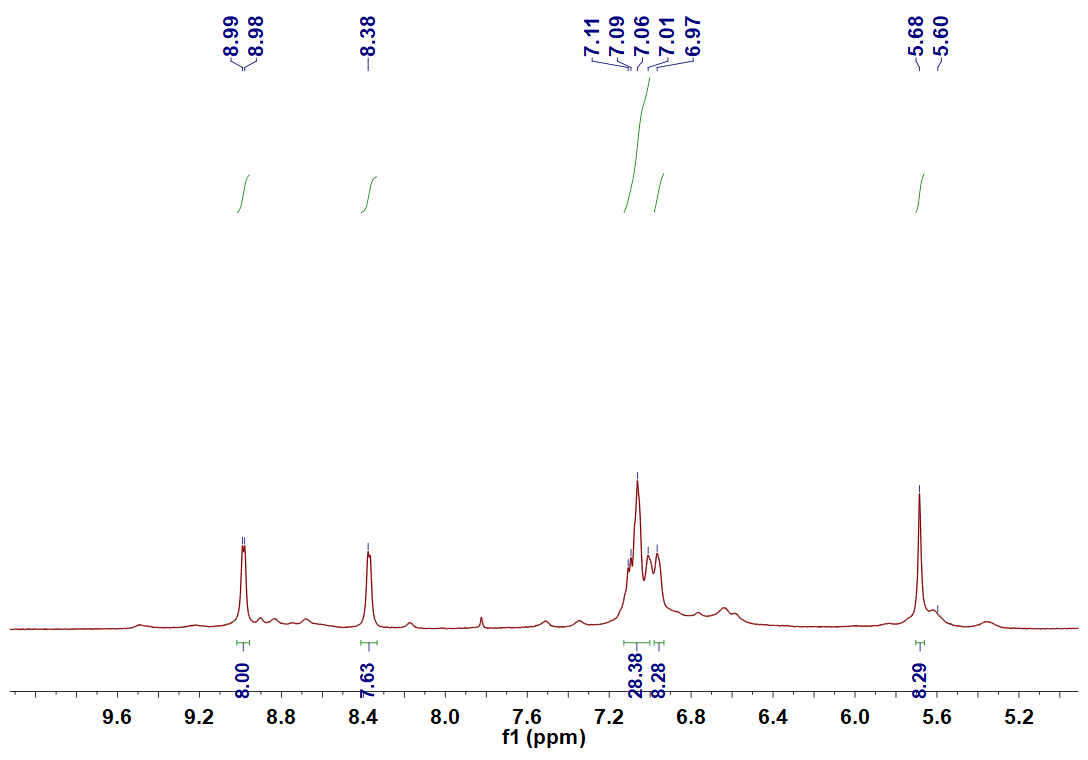


***Fig. S8*** ^1^H NMR spectrum (400 MHz, D_2_O, room temperature) of **TPE-cyc**.

*3. ESI-MS spectrum of* ***TPE-cyc⊃NADPH*** *in H_2_O.*

*
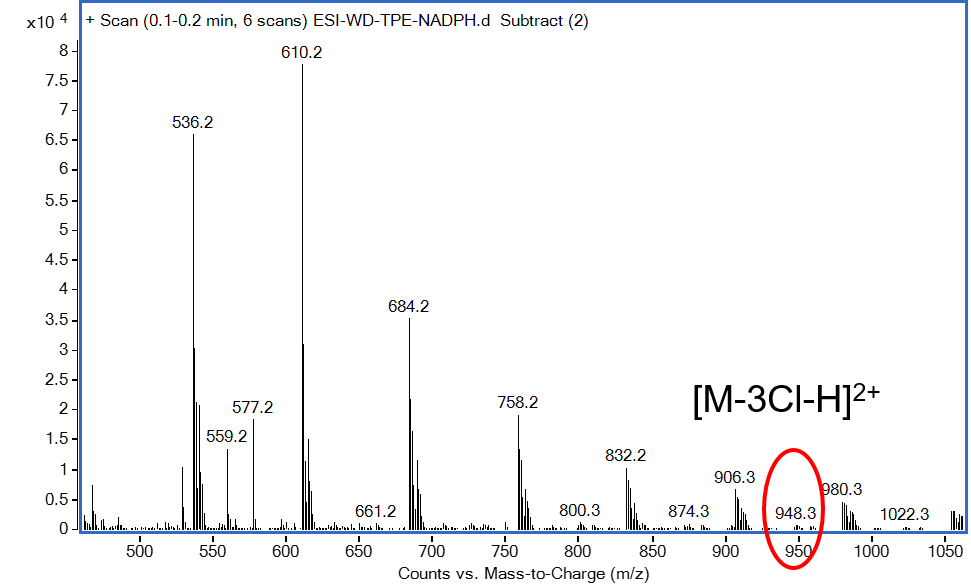
*

***Fig. S9*** *ESI-MS spectrum of* ***TPE-cyc⊃NADPH*** *in H_2_O*.

*4. Job’s plots obtained by recording the absorption maximum of* ***TPE-cyc*** *and NADPH.*

*
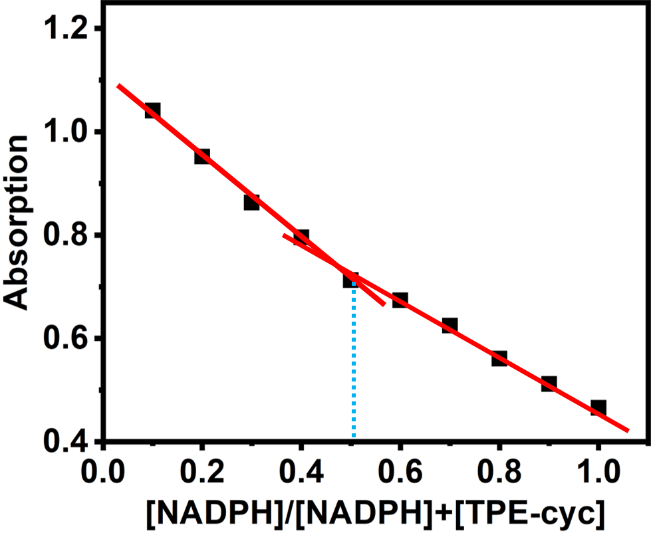
*

***Fig. S10*** *Job’s plots obtained by recording the absorption maximum of* ***TPE-cyc*** *and NADPH*.

*5. Cytotoxicity evaluation.*


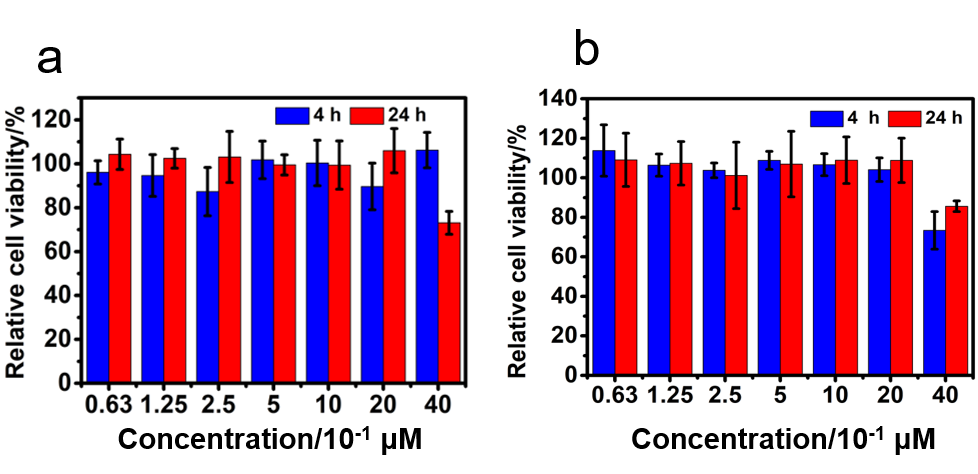


***Fig. S11*** Cytotoxicity of **6** against (a) HeLa cells and (b) U87 cells with different concentrations for 4 h and 24 h.

*6. Fluorescent images of ROS in HeLa cells incubated with* ***TPE-cyc****.*


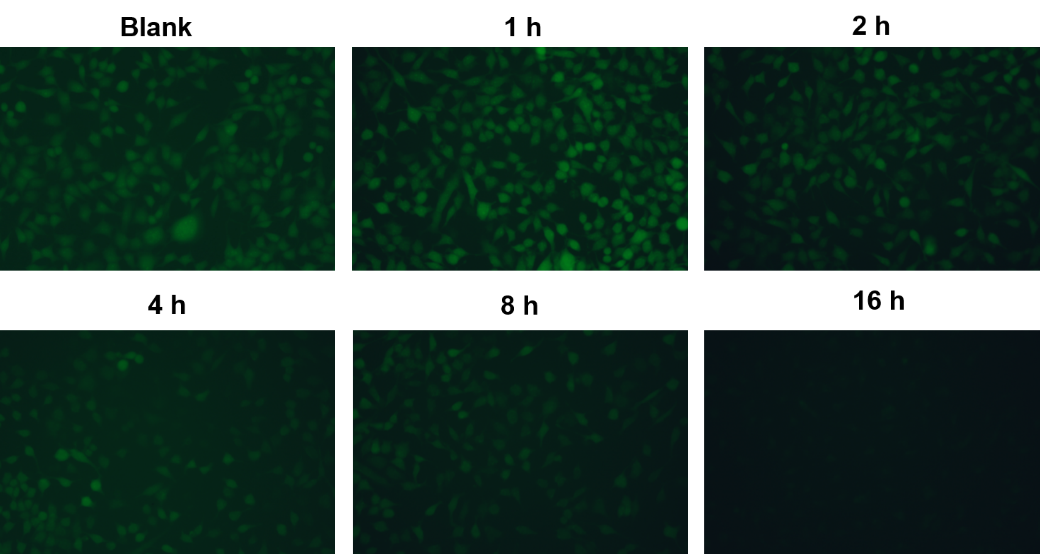


***Fig. S12*** Fluorescent images of ROS in HeLa cells incubated with **TPE-cyc** for different time.


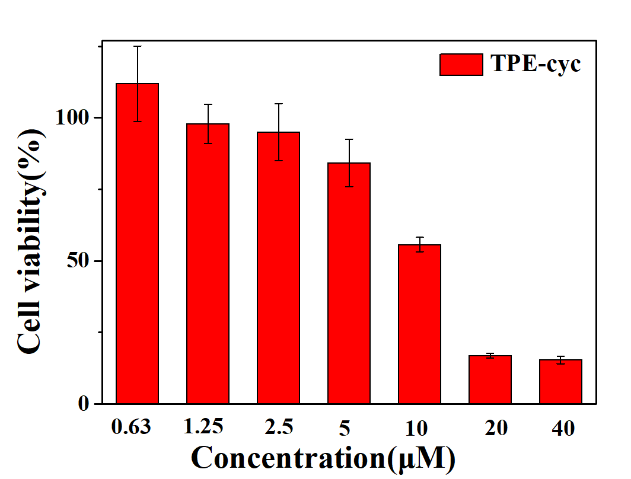


***Fig. S13*** Cytotoxicity of **TPE-cyc** against HUVEC cells with different concentrations for 24 h.

**Cell Cultures.** HeLa cells and U87 cells (human cervical carcinoma, ATCC) were cultured in Dulbecco's modified Eagle's medium (DMEM) containing 10% fetal bovine serum (FBS) and 1% penicillin/streptomycin. The cells were harvested from the cell culture medium by incubating in a trypsin solution (0.5% *w*/*v* in PBS) for 1 min. The cells were centrifuged, and the supernatant was discarded. The cells were resuspended in serum-supplemented DMEM at a concentration of 1 × 10^4^ cells/mL. Cells were cultured at 37 °C and 5% CO_2_.

**Evaluation of Cytotoxicity.** The cytotoxicity of **6** and **TPE-cyc** were evaluated by using MTT assay. HeLa cells or U87 cells were seeded in 96-well plates at 8×10^3^ cells/well, allowed to adhere overnight and incubated with serum-free culture media containing **6** and **TPE-cyc** at a serious of concentrations. After 4 h or 24 h incubation, the media was removed and washed with PBS three times. The cells were incubated in 100 μL DMEM medium containing 0.5 mg/mL MTT reagent for an additional 4 h, 100 μL DMSO was added to each well to dissolve formazan crytal. Eventually, each well was measured using a scanning spectrophotometer (Model 550, Bio-Rad) at a wavelength 570 nm.

**Cellular uptake assay.** HeLa cells or U87 cells were cultured in the chambers at a density of 1 × 10^5^ per well for 24 h. The cells were incubated with **TPE-cyc** at 37 °C for 2 h, 4 h and 8 h respectively, followed by staining with 5 μM Nuclear Green LCS1 for 30 min. The images were taken using a LSM-510 confocal laser scanning microscope (Radiance2100, Bio-Rad) (100 × oil immersion lens).

**Cellular ROS assay.** HeLa cells were planted in six well plates, and the cell density was 2 × 10^5^ cells/well. After 24 h incubation, the **TPE-cyc** in fresh DMEM medium were added and the HeLa cells were incubated for 1 h, 2 h, 4 h and 8 h, respectively. At last, the cells were trypsinized and resuspended in PBS (0.5 mL). Flow cytometry measurements were conducted using a FACSCalibur flow cytometer (BD FACSCalibur). The mean fluorescence signal was determined by counting 10000 events

**Reference**:

S1. L. Cheng, H. Y. Zhang, Y. H. Dong, Y. X. Zhao, Y. Yu and L. P. Cao, *Chem. Commun.*, 2019, **55**, 2372–2375.
